# Long-term clinical and radiological outcome in patients with severe Legg-Calvé-Perthes disease after Chiari pelvic osteotomy: a mean of 14 years follow-up

**DOI:** 10.1177/1120700020988150

**Published:** 2021-02-10

**Authors:** Dietmar Dammerer, Matthias Braito, Peter Ferlic, Gerhard Kaufmann, Juana Kosiol, Rainer Biedermann

**Affiliations:** 1 Department of Orthopaedics and Traumatology, Medical University of Innsbruck, Innsbruck, Austria; 2 Department of Orthopaedics and Traumatology, St Johann in Tirol District Hospital, St. Johann in Tirol, Austria; 3 OFZ Innsbruck, Innsbruck, Austria

**Keywords:** Chiari pelvic osteotomy, Legg-Calvé-Perthes disease, long-term results, salvage

## Abstract

**Introduction:**

The Chiari pelvic osteotomy (CPO) has been recommended as a salvage procedure to improve head coverage in case of hip joint incongruence in paediatric hip disease. In this study, we aimed to assess the long-term results of CPO for severe Legg-Calvé-Perthes disease (LCPD).

**Methods:**

A total of 39 patients who underwent a CPO at our department between 1995 and 2010 were prospectively followed both radiologically (Stulberg classification) and clinically (Harris Hip Score [HHS], conversion into total hip arthroplasty). In this study, we retrospectively reviewed the cases of 12 hips (12 patients, 3 girls, 8 left hips) treated by CPO for severe LCPD (Catterall grade 3 or 4) with hip joint incongruence. Mean follow-up was 14.0 (range 7.6–21.3) years.

**Results:**

Mean age at surgery was 10.2 (range 8.2–17.8) years. Additional femoral osteotomy was performed in 8 patients. A good radiological result (Stulberg I or II) was achieved in 2 patients, a fair result (Stulberg III) in 4 patients, and a poor outcome (Stulberg IV or V) in 6 patients. Mean postoperative HHS averaged 93 (range 65–100) points. An excellent functional outcome (HHS 90–100 points) was achieved in 9 patients. No patient underwent total hip arthroplasty during follow-up. Postoperative limb-length discrepancy was found in 3 patients.

**Conclusions:**

CPO for severe LCPD with hip joint incongruence resulted in good long-term clinical outcome in about ⅔ of our patients after a mean of 14 years. Our results suggest that CPO can still be considered as a salvage joint-conserving procedure in this selected group of younger patients.

## Introduction

There is great variability in the clinical and radiographic expression of Legg-Calvé-Perthes disease (LCPD).^1,2^ LCPD results in a temporary softening of the femoral head, which may further lead to structural abnormities, hip joint incongruence, and secondary hip osteoarthritis. Conservative and operative treatments aim to minimise femoral head deformity to prevent premature osteoarthritis of the hip.^3^ Main goals of LCPD treatment are femoral head containment and maintenance of hip motion.^3^

A variety of different surgical treatment options have been described in the literature and can be classified as proximal femoral procedures,^4,5^ pelvic procedures,^5–9^ or combined procedures.^10^ From a biomechanical view, correction of the acetabulum is the preferred procedure.^11^

Already in 1952 Prof. Karl Chiari performed the first Chiari pelvic osteotomy (CPO) and reported encouraging short-term results.^12,13^ Initially, Chiari limited the indications to subluxated and dislocated hips and claimed that his method provides stable coverage of the femoral head and improved biomechanics of the treated hip.^14^ In the current literature CPO and shelf-acetabuloplasty are mainly considered salvage procedures for the treatment of LCPD with hip joint incongruence in adolescents and young adults.^15–21^

In this study, we reviewed the results of a selected group of patients with incongruent hips resulting from severe LCPD and treated by CPO at our department. We aimed to assess long-term radiographic and clinical outcome of patients with severe LCPD after CPO.

## Methods

The local ethics committee approved the study protocol and written informed consent was obtained from all patients.

We identified all patients at our institution who had undergone CPO from March 1995 to June 2010. CPO was performed in selected cases of LCPD with inadequate femoral head coverage. In this context, CPO was preferred over varus femoral osteotomy in cases with limited hip abduction (<30°). However, according to our previous institutional practice, we did not restrict CPO to cases with hip joint incongruence at that time. Additional femoral valgus extension osteotomy was performed in case of LCPD with hinge abduction (impingement of the extruded superolateral portion of the femoral head against the lateral acetabulum on arthrography).

**Figure 1.**
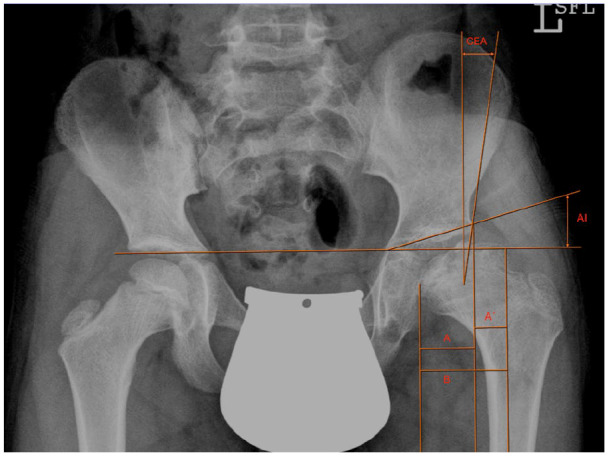
Preoperative radiograph and radiographic measurements of a 9-year-old boy with severe LCPD of the left hip with inadequate femoral head coverage and hip joint incongruence. AI, acetabular index; CEA, centre-edge angle; FHC, femoral head coverage percentage = A/B × 100; RI, Reimer’s index = A’/B × 100. AI, acetabular index; CEA, centre-edge angle; FHC, femoral head coverage percentage = A/B × 100; RI, Reimer’s index = A’/B × 100.

**Figure 2.**
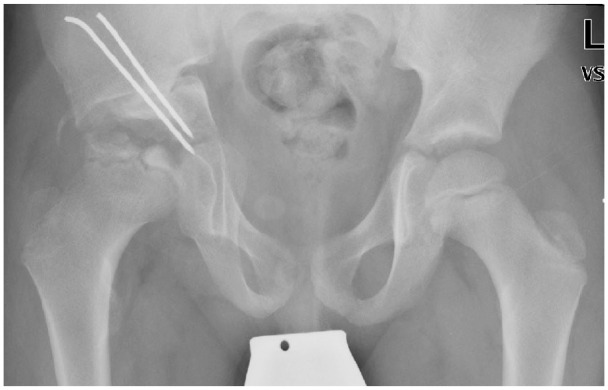
Postoperative radiograph of a 10-year-old boy after Chiari pelvic osteotomy for severe LCPD of the right hip.

**Figure 3.**
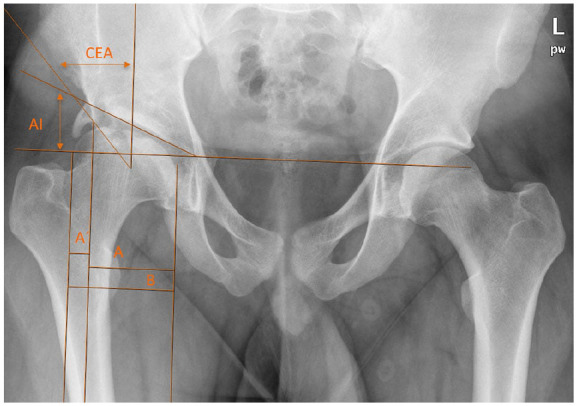
Postoperative radiograph and radiographic measurements 10 years after Chiari pelvic osteotomy for severe LCPD of the right hip. AI, acetabular index; CEA, centre-edge angle; FHC, femoral head coverage percentage = A/B × 100; RI, Reimer’s index = A’/B × 100.

A total number of 39 hips (23 left hips, 16 right hips) in 39 consecutive patients (10 women, 29 men) were retrospectively identified. Catteral grades of these 39 consecutive patients were as follows: Catteral 1 (*n* = 5), Caterall 2 (*n* = 6), Catteral 3 (*n* = 12), and Catteral 4 (*n* = 11). Preoperative radiographs were missing in 5 cases. In 4 patients hip disease was bilateral, but CPO was performed only on the more affected side, while the contralateral hip was managed conservatively. All patients were routinely followed by clinical and radiological examinations (Figures 1–3).

In order to determine the value of CPO for severe cases of LCPD (Catterall grade 3 or 4), we retrospectively reviewed the cases of 12 hips (12 patients, 3 girls, 8 left hips) with hip joint incongruence in this study. In addition to our routine follow-up examinations, all patients were invited by telephone to participate in a separate study visit at our institution. This study visit included completion of Harris Hip Score (HHS) questionnaires, a physical examination, and a review of the latest x-rays. 1 patient was unable to follow our invitation for the follow up-examination in our hospital. Thus, 11 patients completed the follow-up study examination. Mean follow-up was 14.0 (range 7.6–21.3) years. Preoperative x-rays were not available for retrospective analysis in 3 patients. Patients’ characteristics are shown in Table 1.

**Table 1.** Pre- and postoperative patients’ radiological data.

Patient	Sex	Side	Age at surgery (years)	Femoral osteotomy	Herring	Catteral	CEA (°)	AI (°)	RI (%)	FHC (%)	Stulberg	CEA (°)	AI (°)	RI (%)	FHC (%)
					**Preoperative**						**postoperative**				
1	M	left	8.5	–	C	IV	13.7	14	37	37	3	30.6	19.2	32	47
2	M	left	10.3	Varus	C	III	–	–	–	–	4	44.3	17.4	12	76
3	M	right	9.2	–	C	IV	24.2	17.7	29	51	1	34.4	14.1	20	66
4	M	left	8.4	Valgus	C	IV	38.2	27.8	15	71	5	45.2	12.3	12	78
5	M	left	17.8	Valgus	C	III	−18.6	27.8	56	21	4	18.5	18.6	38	39
6	M	right	9.3	Valgus	C	IV	26.4	11.3	31	47	3	47.9	11.1	12	78
7	M	left	8.2	Valgus	B	III	–	–	–	–	1	37.9	19.2	8	83
8	M	left	9.3	Varus	C	IV	30.9	7.7	0	100	3	34.8	17	22	59
9	M	right	8.4	–	B	III	–	–	–	–	3	25.5	25.1	32	47
10	F	left	13.4	–	C	III	10	18.8	45	30	4	44.4	14.3	16	69
11	F	left	10.9	Valgus	C	III	29.7	10	6	83	5	37.7	15.6	0	100
12	F	right	9.1	Valgus	C	III	47.8	9.4	0	100	4	37.1	17.2	5	91

CEA, centre-edge angle; AI, acetabular index; RI, Reimer’s index; FHC, femoral head coverage.

The operative technique of the Chiari pelvic osteotomy is described elsewhere.^12^ The basic elements comprise a semi-circular osteotomy in the sagittal plane and an ascending straight osteotomy in the frontal plane of the ilium.^12,13,18^ Preoperative hip arthrography was routinely performed. A single leg-hip spica cast was used for postoperative immobilisation in younger patients for 4–6 weeks followed by full weight-bearing and physical therapy.

Follow-up examinations were performed by 3 authors. The HHS was utilised to assess clinical outcome.^22^ Furthermore, all patients were asked about their actual pain level using the numeric rating scale (NRS; 0–10; 0 = no pain; 10 = pain as intense as you can imagine). Hip range of motion was measured using a goniometer. Leg-length discrepancy was assessed using standing blocks.

For radiographic evaluation we used anteroposterior films of the pelvis taken before and after osteotomy and at follow-up examination. Acetabular Index (AI) and Wiberg’s Centre-edge Angle (CEA) measurements were taken as references for the acetabulum shape.^23^ The AI was measured as the angle formed between the lateral margin of the acetabular roof and the inferior aspect of the pelvic teardrop and a horizontal line between the inferior aspect of both pelvic teardrops. The CEA was formed by a vertical line from the centre of the femoral head and a line from the centre of the femoral head to the lateral edge of the acetabulum. The femoral head cover percentage (FHC) and Reimer’s Index (RI) were calculated as the horizontal distance of the lateral femoral head covered (A)/uncovered (A’) by the acetabulum divided by the total horizontal width of the femoral head (B). Both indices were expressed as a percentage (Figures 1 and 3).^24^ Catterall and Herring classifications were used to preoperatively assess the stage of femoral head involvement.^2,25^ Stulberg classification was applied to evaluate the femoral head condition in radiographic outcomes and summarised as good (I/II), fair (III) or poor (IV/V) postoperatively.^26,27^

### Statistical analysis

The statistical analysis was conducted in a purely descriptive manner with IBM SPSS Statistics 22 (IBM Corporation, Armonk, NY, USA). Variables on a nominal scale are reported as absolute values and percentages, variables on an ordinal scale as median and minimum/maximum, variables on an interval scale with mean and standard deviation. The non-parametric Shapiro-Wilk test was used to test for normality.

## Results

CPO was performed in 12 patients (12 hips, nine boys and three girls). Mean age at presentation at our department was 9.1 (range 5.9–17.0) years and mean age at operation was (range 8.2–17.8) 10.2 years, respectively. In 3 cases, a concurrent femoral valgus osteotomy was applied via a lateral approach to obtain concentric reduction of the femoral head within the acetabulum. In 5 cases, femoral valgus (3 hips) or varus (2 hips) osteotomy was performed at an average of 1.5 years (range 0.3–2.3) before CPO. Screw removal was carried out routinely at 0.9 (range 0.3–1.6) years after CPO. 2 of the 3 females had normal spontaneous vaginal delivery after CPO.

### Radiological findings

According to the preoperative Herring and Catterall classifications, 2 hips were graded Herring B, 10 hips Herring C, 7 hips Catterall III, and 5 hips Catterall IV. A good postoperative radiographic outcome according to the Stulberg classification was achieved in 2 patients (Stulberg I/II), a fair outcome (Stulberg III) in 4 patients, and a poor postoperative outcome (Stulberg IV/V) in 6 patients.

Femoral head coverage improved in all patients after CPO. Mean pre- and postoperative acetabular Index (AI) was 16.1° (range 7.7°−27.8°) and 16.8° (range 11.1°−25.1°), Wiberg’s Centre-edge Angle (CEA) averaged 22.5° (range −18.6°−47.8°) and 36.5° (range 18.5°−47.9°), mean femoral head cover percentage (FHC) 60% (range 20–100%) and 69% (range 39–100%), and mean postoperative Reimer’s Index (RI) 24% (range 0–56%) and 17% (range 0–38%). Radiological results of each patient are shown in Table 1.

### Clinical findings

Mean postoperative Harris Hip Score averaged 93 (range 65–100) points. According to the HHS, an excellent functional outcome (90–100 points) was achieved in 9 patients, a good functional outcome in 1 patient, and a poor functional outcome (HHS <70 points) in 1 patient. Mean NRS for pain at the time of latest follow-up was 1 (0–5). No patient underwent total hip arthroplasty during follow-up.

Postoperative limb-length discrepancy was found in 3 patients and averaged 1.3 cm (range 1.0–1.8). All 3 limb-length discrepancies were caused by lengthening of the operated limb. 9 patients showed no limb–length discrepancy.

Postoperative range of motion of the operated hip averaged as follows: external rotation 30° (range 0–80°), internal rotation 20° (range 0–50°), abduction 30° (range 10–40°), adduction 25° (range 20–30°), and flexion 100° (range 90–110). None of the patients showed a positive Trendelenburg’s sign postoperatively (Table 2).

**Table 2.** Postoperative patients’ clinical outcome.

Patient	FU (years)	HHS (points)	NRS	ER (°)	IR (°)	ABD (°)	ADD (°)	FLEX (°)	LLD (cm)
1	16.1	–	–	–	–	–	–	–	–
2	12.8	98	0	0	40	20	30	100	1.8
3	14.6	100	0	30	30	40	20	110	–
4	10.4	96	3	80	0	20	20	110	1.0
5	9.5	65	5	25	0	30	30	90	–
6	7.6	98	0	50	50	40	30	110	–
7	21.3	95	0	5	15	30	20	110	1.0
8	20.2	95	0	35	40	20	30	90	–
9	15.2	92	2	30	0	40	20	100	–
10	19.3	97	0	20	30	20	30	110	–
11	10.3	96	1	40	10	40	20	100	–
12	10.8	87	0	20	0	10	20	100	–

FU, follow-up; HHS, Harris Hip Score; 0–100 points; NRS, numeric rating scale for pain; 0–10; ER, external rotation; IR, internal rotation; ABD, abduction; ADD, adduction; FLEX, flexion; LLD, limb-length discrepancy.

## Discussion

Our study findings show good long-term clinical outcome at 14 years after CPO for severe LCPD with hip joint incongruence in about ⅔ of our patients. HHS averaged 93 points, showing only minor impairments for activities of daily living. An excellent functional clinical outcome (HHS 90–100 points) was achieved in ¾ (9 of 12) of patients. These clinical findings are in contrast to the radiographic outcomes, as only 2 patients who were graded as Herring B (Caterall 3) and underwent surgery before the age of 10 years exhibited a good postoperative result according to the Stulberg classification.

Long-term results following CPO have already been reported in the literature.^28–30^ Most of those reports deal with the outcome in patients with dysplastic hips and poor joint congruency.^31^ The best treatment for patients with LCPD and hinge abduction continues to be controversial and the role of CPO is still under debate. To our best knowledge only few authors have investigated outcome following CPO in patients with severe LCPD.^3,32–35^

From the given literature it is seen that the most important factors for successful surgical treatment in severe LCPD are congruency of the hip joint and femoral head sphericity.^32,35–37^ Different types of surgical procedures for the treatment of LCPD are suggested in the literature. CPO is generally considered to be a salvage procedure for the treatment of LCPD when other osteotomies seem inappropriate.^3^ Bennett et al.^15^ reported in their study with severe cases of LCPD and incongruent hip joints that most pelvic osteotomies like the Salter-Innominate osteotomy are contraindicated. Klisic et al.^32^ concluded that CPO is needed in hips where neither non-operative treatment nor femoral or innominate redirectional osteotomy could be expected to be successful. Reinker^38^ mentioned that hinge abduction should be considered a contraindication to containment by redirectional pelvic or femoral varus osteotomy. For patients in whom hinge abduction is suspected, a shelf arthroplasty or CPO is recommended, but if a reasonably sized shelf would not adequately cover the femoral head, a Chiari osteotomy would be a valuable alternative.^38^ We do not perform shelf arthroplasty and therefore have no experience with this procedure at our institution.

Lack et al.^35^ stated that CPO might delay the clinical effects of hip osteoarthritis for years in patients with LCPD. This finding was also supported by other studies that indicated that the degenerative process might be slowed as a result of the favourable biomechanical properties of CPO.^18,35–37^ This might be explained by reduction of the joint pressure due to increased weight-bearing surface, shortening of the lever arm of the body weight, and centralisation of the femoral head within its muscle cuff.^35–37^ We cannot support the view of Kamegaya et al.^39^ that CPO leads to a medialisation of the hip centre. According to Stulberg et al.^26^, the most important factors for successful surgical treatment of severe LCPD are congruency of the hip joint and femoral head sphericity. However, for patients with an incongruent hip joint and/or hinge abduction phenomenon CPO could be a valuable alternative. In our study, we demonstrate a significant improvement in femoral head coverage and a good clinical outcome in about ⅔ of such patients.

CPO has been recommended mainly for adolescents and young adults.^40^ Long-term survival of the hip joint was related to age at operation for hip dysplasia.^28,29,41,42^ Our results indicate that children and adolescents with LCPD aged between 8 and 18 years undergoing CPO can achieve good functional results. This might be explained by the improved coverage of the femoral head after CPO, which hypothetically contributes to the epiphyseal growth, and promotes remodelling of the femoral head. Reddy and Morin^3^ reported good results after CPO in children with an average age at surgery of 8.5 years. Crutcher and Staheli^43^ found a 50% improvement in femoral head sphericity in young children classified as Caterall group III and IV cases after combined Salter innominate and varus femoral osteotomies. Consequently, Canale et al.^6^ report poor remodelling in all cases operated at late stage LCPD. Also Windhager et al.^29^ found a poorer outcome with increasing age at the time of operation. In contrast, in a study by Cahuzac et al.^16^, younger patients were seen to have a poorer outcome. In summary, the question as to the right age for an LCPD patient with an incongruent hip joint to undergo CPO remains unanswered.

We acknowledge the following limitations of our study. Foremost, the relatively small number of patients may inhibit adequate analysis of significant risk and prognostic factors of CPO in LCPD. Furthermore, the complete dataset was not available for all patients and patient specifications were inhomogeneous at the time of surgery. This, however, is mostly the case in paediatric orthopaedic surgery with rare diseases and heterogeneous approaches. Furthermore, the Harris hips score used to assess functional outcome was developed for adults with hip osteoarthritis. Therefore, it may not be sufficiently sensitive in a population of young adults with the deformity prior to the onset of osteoarthritis.

In conclusion, the best treatment for LCPD patients with hinge abduction continues to be controversial. The Chiari pelvic osteotomy is a salvage procedure, effective for improving hip coverage and diminishing joint pressure. We therefore believe that the Chiari pelvic osteotomy can be considered a salvage procedure for children with severe cases of LCPD with incongruent hip joints and hinge abduction, when containment procedures are contraindicated.

## References

[1] Herring JA Kim HT Browne R. Legg-Calve-Perthes disease. Part I: classification of radiographs with use of the modified lateral pillar and Stulberg classifications. J Bone Joint Surg Am 2004; 86: 2103–2120. 15466719

[2] Catterall A. The natural history of Perthes’ disease. J Bone Joint Surg Br 1971; 53: 37–53. 5578764

[3] Reddy RR Morin C. Chiari osteotomy in Legg-Calve-Perthes disease. J Pediatr Orthop B 2005; 14: 1–9. 15577300 10.1097/01202412-200501000-00001

[4] Fulford GE Lunn PG Macnicol MF. A prospective study of nonoperative and operative management for Perthes’ disease. J Pediatr Orthop 1993; 13: 281–285. 8496357 10.1097/01241398-199305000-00001

[5] Wang L Bowen JR Puniak MA , et al. An evaluation of various methods of treatment for Legg-Calve-Perthes disease. Clin Orthop Relat Res 1995; 314: 225–233. 7634639

[6] Canale ST D’Anca AF Cotler JM , et al. Innominate osteotomy in Legg-Calve-Perthes disease. J Bone Joint Surg Am 1972; 54: 25–40. 4559945

[7] Grudziak JS Ward WT. Dega osteotomy for the treatment of congenital dysplasia of the hip. J Bone Joint Surg Am 2001; 83: 845–854. 11407792 10.2106/00004623-200106000-00005

[8] Paterson DC Leitch JM Foster BK. Results of innominate osteotomy in the treatment of Legg-Calve-Perthes disease. Clin Orthop Relat Res 1991; 266: 96–103. 2019074

[9] Steel HH. Triple osteotomy of the innominate bone. J Bone Joint Surg Am 1973; 55: 343–350. 4572223

[10] Chakirgil GS, Isitman AT and Ceten I. Double osteotomy operation in the surgical treatment of coxa plana disease. Orthopedics 1985; 8: 1495–1504. 3832036 10.3928/0147-7447-19851201-10

[11] Westhoff B Martiny F Krauspe R. [Current treatment strategies in Legg-Calve-Perthes disease]. Orthopade 2013; 42: 1008–1017. 24201830 10.1007/s00132-012-2048-y

[12] Chiari K. [Pelvic osteotomy in hip arthroplasty]. Wien Med Wochenschr 1953; 103: 707–709. 13103071

[13] Chiari K. [Results of pelvic osteotomy as of the shelf method acetabular roof plastic]. Z Orthop Ihre Grenzgeb 1955; 87: 14–26. 13312490

[14] Chiari K. [Pelvic osteotomy in the treatment of coxarthrosis]. Beitr Orthop Traumatol 1968; 15: 163–168. 5685082

[15] Bennett JT Mazurek RT Cash JD. Chiari’s osteotomy in the treatment of Perthes’ disease. J Bone Joint Surg Br 1991; 73: 225–228. 2005144 10.1302/0301-620X.73B2.2005144

[16] Cahuzac JP Onimus M Trottmann F , et al. Chiari pelvic osteotomy in Perthes disease. J Pediatr Orthop 1990; 10: 163–166. 2312693

[17] Calvert PT August AC Albert JS , et al. The Chiari pelvic osteotomy. A review of the long-term results. J Bone Joint Surg Br 1987; 69: 551–555. 3611157 10.1302/0301-620X.69B4.3611157

[18] Chiari K. Medial displacement osteotomy of the pelvis. Clin Orthop Relat Res 1974; 98: 55–71. 10.1097/00003086-197401000-00008 4817245

[19] De Waal Malefijt MC Hoogland T Nielsen HK. Chiari osteotomy in the treatment of congenital dislocation and subluxation of the hip. J Bone Joint Surg Am 1982; 64: 996–1004. 7118987

[20] Reynolds DA. Chiari innominate osteotomy in adults. Technique, indications and contra-indications. J Bone Joint Surg Br 1986; 68: 45–54. 3941141 10.1302/0301-620X.68B1.3941141

[21] Willett K Hudson I Catterall A. Lateral shelf acetabuloplasty: an operation for older children with Perthes’ disease. J Pediatr Orthop 1992; 12: 563–568. 1517413

[22] Harris WH. Traumatic arthritis of the hip after dislocation and acetabular fractures: treatment by mold arthroplasty. An end-result study using a new method of result evaluation. J Bone Joint Surg Am 1969; 51: 737–755. 5783851

[23] Wiberg G. Studies on dysplastic acetabulum and congenital subluxation of the hip joint with special reference to the complication of osteoarthritis. Acta Chir Scand 1939; 83: 58.

[24] Reimers J. The stability of the hip in children. A radiological study of the results of muscle surgery in cerebral palsy. Acta Orthop Scand Suppl 1980; 184: 1–100. 6930145 10.3109/ort.1980.51.suppl-184.01

[25] Herring JA Neustadt JB Williams JJ , et al. The lateral pillar classification of Legg-Calve-Perthes disease. J Pediatr Orthop 1992; 12: 143–150. 1552014 10.1097/01241398-199203000-00001

[26] Stulberg SD Cooperman DR Wallensten R. The natural history of Legg-Calve-Perthes disease. J Bone Joint Surg Am 1981; 63: 1095–1108. 7276045

[27] Neyt JG Weinstein SL Spratt KF , et al. Stulberg classification system for evaluation of Legg-Calvé-Perthes disease: intra-rater and inter-rater reliability. J Bone Joint Surg Am 1999; 81: 1209–1216. 10505517 10.2106/00004623-199909000-00002

[28] Kotz R Chiari C Hofstaetter JG , et al. Long-term experience with Chiari’s osteotomy. Clin Orthop Relat Res 2009; 467: 2215–2220. 19521741 10.1007/s11999-009-0910-y PMC2866931

[bibr29-1120700020988150] Windhager R Pongracz N Schönecker W , et al. Chiari osteotomy for congenital dislocation and subluxation of the hip. Results after 20 to 34 years follow-up. J Bone Joint Surg Br 1991; 73: 890–895. 1955430 10.1302/0301-620X.73B6.1955430

[30] Piontek T Szulc A Glowacki M , et al. Distant outcomes of the Chiari osteotomy 30 years follow up evaluation. Ortop Traumatol Rehabil 2006; 8: 16–23. 17603450

[31] Ito H Tanino H Yamanaka Y , et al. The Chiari pelvic osteotomy for patients with dysplastic hips and poor joint congruency: long-term follow-up. J Bone Joint Surg Br 2011; 93: 726–731. 21586768 10.1302/0301-620X.93B6.26178

[32] Klisic P Bauer R Bensahel H , et al. Chiari’s pelvic osteotomy in the treatment of Legg-Calvé-Perthes disease. Bull Hosp Jt Dis Orthop Inst 1985; 45: 111–118. 3000490

[bibr33-1120700020988150] Kerschbaumer F Bauer R. [Innominate osteotomy (Chiari) for the treatment of coxa magna–preliminary results of 17 patients (author’s transl)]. Arch Orthop Unfallchir 1977; 87: 137–149. 843288 10.1007/BF00415202

[bibr34-1120700020988150] Cahuzac JP Du Boullay C Onimus M , et al. [Perthes’ disease treated by Chiari pelvic osteotomy (author’s transl)]. Rev Chir Orthop Reparatrice Appar Mot 1981; 67: 133–139. 6453402

[35] Lack W Feldner-Busztin H Ritschl P , et al. The results of surgical treatment for Perthes’ disease. J Pediatr Orthop 1989; 9: 197–204. 2925854

[bibr36-1120700020988150] Lack W Windhager R Kutschera HP , et al. Chiari pelvic osteotomy for osteoarthritis secondary to hip dysplasia. Indications and long-term results. J Bone Joint Surg Br 1991; 73: 229–234. 2005145 10.1302/0301-620X.73B2.2005145

[37] Chairi K Endler M Hackel H. [Treatment of coxa magna in Perthes disease by pelvic osteotomy (author’s transl)]. Arch Orthop Trauma Surg 1978; 91: 183–190. 666547 10.1007/BF00379749

[38] Reinker KA. Shelf and/or reduction and containment surgery. Orthop Clin North Am 2011; 42: 355–359, vii. 21742147 10.1016/j.ocl.2011.03.003

[39] Kamegaya M Shinada Y Moriya H , et al. Acetabular remodelling in Perthes’ disease after primary healing. J Pediatr Orthop 1992; 12: 308–314. 1572993 10.1097/01241398-199205000-00006

[40] Vukasinovic Z Spasovski D Slavkovic N , et al. Chiari pelvic osteotomy in the treatment of adolescent hip disorders: possibilities, limitations and complications. Int Orthop 2011; 35: 1203–1208. 20878156 10.1007/s00264-010-1126-1 PMC3167444

[41] Macnicol MF Lo HK Yong KF. Pelvic remodelling after the Chiari osteotomy. A long-term review. J Bone Joint Surg Br 2004; 86: 648–654. 15274258 10.1302/0301-620x.86b5.14653

[42] Rozkydal Z Kovanda M. Chiari pelvic osteotomy in the management of developmental hip dysplasia: a long term follow-up. Bratisl Lek Listy 2003; 104: 7–13. 12830990

[43] Crutcher JP Staheli LT. Combined osteotomy as a salvage procedure for severe Legg-Calve-Perthes disease. J Pediatr Orthop 1992; 12: 151–156. 1552015 10.1097/01241398-199203000-00002

